# Gene‐Smoking Interaction in Insulin Sensitivity and β‐Cell Function Among Normal Glucose Tolerance Individuals

**DOI:** 10.1111/1753-0407.70131

**Published:** 2025-07-28

**Authors:** Pan Gu, Shuai Zheng, Sijie Zhang, Jie Yuan, Hao Hong, Jinglan Dai, Jingyi Zhao, Kuanfeng Xu, Tao Yang, Qi Fu, Sipeng Shen, Hao Dai

**Affiliations:** ^1^ Department of Biostatistics Center for Global Health, School of Public Health, Nanjing Medical University Nanjing China; ^2^ Department of Endocrinology and Metabolism The First Affiliated Hospital With Nanjing Medical University Nanjing China; ^3^ Jiangsu Key Lab of Cancer Biomarkers, Prevention and Treatment, Jiangsu Collaborative Innovation Center for Cancer Personalized Medicine Nanjing Medical University Nanjing China

**Keywords:** gene–environment interaction, insulin sensitivity, smoking, β‐Cell function

## Abstract

**Objective:**

To identify genetic loci that exhibit potential interactions with smoking status on insulin sensitivity and islet β‐cell function within normal glucose tolerance (NGT) populations.

**Methods:**

All participants underwent an OGTT to confirm NGT status, followed by assessments of insulin sensitivity and β‐cell function. Analyses were performed in NGT participants from Nanjing (*N* = 4808) and Jurong (*N* = 508) for discovery and validation, respectively. Smoking status was categorized into nonsmokers and smokers. After excluding ineligible individuals, a two‐stage genome‐wide interaction association analysis (GWIS) was conducted in NGT individuals, with the discovery phase (*N* = 1377) identifying gene–environment interactions and the validation phase (*N* = 485) confirming significant loci. Subsequent analyses included stratified analysis and expression quantitative trait locus (eQTL) colocalization.

**Results:**

GWIS identified ten SNPs in three loci, including rs4713207 (*OR14J1*, *P*_meta_ = 3.95 × 10^−8^) for insulin resistance, rs17708475 (*NKAIN2, P*_meta_ = 4.83 × 10^−8^) for insulin sensitivity, and rs201613 (*MYH3, P*_meta_ = 1.05 × 10^−8^) for disposition index. Stratified analyses revealed differential effects of smoking across genotypes at these loci. Specifically, smoking was associated with increased insulin resistance in rs4713207 homozygotes (*p* = 2.15 × 10^−5^), while an opposite effect was observed in wild‐type individuals (*p* = 0.022). Colocalization analysis indicated that the smoking‐related interaction near rs4713207 is driven by a shared causal variant influencing *HCG4* (PP.H4 = 0.70) and *ZNF311* (PP.H4 = 0.74) expression in the pancreas.

**Conclusions:**

Our findings reveal gene‐smoking interactions that affect insulin sensitivity and β‐cell function, providing new insights into the heterogeneity of metabolic phenotypes and advancing personalized risk assessment.

## Graphical abstract

Gene‐smoking interactions in insulin sensitivity and β‐cell function in normoglycemic individuals.

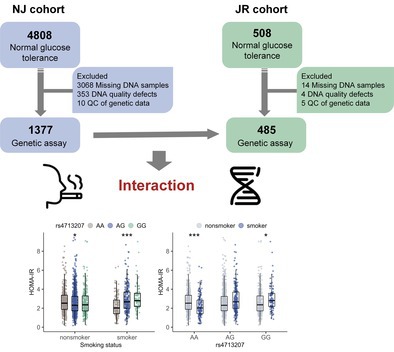

**Summary**

- Variants in OR14J1, NKAIN2, and MYH3 exhibited significant gene‐smoking interactions, influencing key factors of diabetes: insulin sensitivity and β‐cell function.
- eQTL colocalization analysis suggested that rs4713207 affected insulin resistance via ZNF311 and HCG4 expression in the pancreas, while rs201613 was linked to β‐cell function through CTC‐297 N7.1 in the spleen.
- Our findings provide novel insights into the metabolic phenotype variability and contribute to personalized risk evaluation.

## 1 Introduction

Cigarette smoking contributes substantially to the burden of disease, posing significant public health challenges worldwide [1]. Despite global efforts to fight the tobacco epidemic, cigarette smoking remains a key avoidable cause of morbidity and mortality [2]. A large body of research consistently highlights cigarette smoking as a risk factor for diabetes and other metabolic diseases [3, 4]. Smoking is also associated with higher hemoglobin A1c (HbA1c) concentrations [5], and nicotine, the major bioactive substance in cigarettes, has been demonstrated to directly influence glucose homeostasis [6, 7].

Insulin resistance and islet β‐cell dysfunction constitute the two fundamental pathological mechanisms underlying type 2 diabetes (T2D). Previous studies have shown that smokers tend to experience higher insulin resistance than nonsmokers [8], and smoking cessation has been linked to improved insulin sensitivity [9]. Nevertheless, some cross‐sectional studies have suggested that smoking is not associated with insulin resistance in individuals without diabetes [10, 11]. Additionally, the relationship between smoking and β‐cell function remains inconclusive, with studies reporting conflicting results [12, 13]. The heterogeneity among these studies may be attributed to individual differences, shaped by factors such as genetic predisposition, lifestyle behaviors, and health status. Genetic studies on smoking behavior and T2D have independently identified hundreds of loci associated with each of these traits [14, 15, 16, 17]. However, no genome‐wide interaction studies GWIS to date have been conducted to identify genetic loci that modify the relationships between smoking and the pathological mechanisms of T2D.

Therefore, to elucidate the interaction between genetic factors and smoking in relation to insulin sensitivity and islet β‐cell function, we first assessed the association between smoking status and the two core mechanisms of T2D among individuals with NGT. Subsequently, we identified potential interactions between smoking and genetic variants through GWIS to explore whether individuals with different genotypes present divergent responses to smoking status.

## 2 Methods

### 2.1 Study Population

We recruited volunteers with NGT (fasting plasma glucose [FPG] < 6.1 mmol/L and plasma glucose at 120 min [PG120] < 7.8 mmol/L) in Nanjing City (NJ cohort) and Jurong City, Zhenjiang (JR cohort) through community poster advertisements and telephone broadcasts, as previously described [18, 19]. All participants were of Chinese Han ethnicity and received health history assessments along with general health examinations. Glucose metabolism status was determined using an OGTT after a 12‐h fast. Venous blood samples were collected at fasting, 30, and 120 min to measure glucose and insulin levels. Serum insulin levels were quantified via radioimmunoassay (Iodine [125 I] Insulin Radioimmunoassay Kit, Beijing North Institute of Biological Technology). Plasma glucose was measured with the hexokinase method (AU5400, Olympus). Serum triglycerides (TG), serum creatinine (Cr), total cholesterol (TC), low‐density lipoprotein cholesterol (LDL‐C), and high‐density lipoprotein cholesterol (HDL‐C) were measured using chemiluminescence methods with an auto‐analyzer (Modular E170, Roche). A history of hypertension, as well as cardiovascular and cerebrovascular diseases (CVD), was defined by either physician diagnosis or relevant medication use. A family history of diabetes was recorded if any immediate relatives were diagnosed with the condition.

### 2.2 Assessment of Smoking and Phenotypes

Smoking status was categorized into two groups: individuals classified as smokers, including current or former smokers at baseline, and those classified as nonsmokers, defined as individuals with no history of smoking, either past or present.

Insulin resistance was estimated utilizing HOMA‐IR, computed as follows: insulin (mIU/L) × glucose (mmol/L)/22.5 [20]. The Matsuda insulin sensitivity index (ISIc) was employed to evaluate peripheral insulin sensitivity, calculated as 10 000/sqrt [(G0 × INS0) × ($\bar{G}$ × $\;\bar{\mathrm{INS}}$)], where G0 and INS0 represent fasting plasma glucose and serum insulin, $\bar{G}$ and $\bar{\mathrm{INS}}$ represent the average plasma glucose (mg/dL) and serum insulin (mIU/L) levels during the OGTT, respectively [21]. The liver insulin resistance index (LIRI) was calculated using the area under the curve (AUC) for blood glucose from 0 to 30 min of the OGTT multiplied by the AUC for insulin [22].

HOMA‐B was employed to assess β‐cell function as previously described [23]. Insulinogenic index (IGI) was estimated as the change in insulin divided by the change in glucose from 0 to 30 min (ΔI0–30/ΔG0–30) [20]. Corrected insulin response (CIR) was calculated as follows: CIR = [(I30/6.945) × 100]/G30 × (G30–3.89) [24]. Early‐phase insulin secretion was quantified as the ratio of the insulin AUC (mIU/L) to glucose AUC (mmol/L) during the first 30 min of an OGTT, termed INSR30 (InsAUC30/GluAUC30). Similarly, InsAUC120/GluAUC120 (referred to as INSR120) served as a marker for total insulin secretion [25]. Disposition indexes (DI), including DI‐CIR (CIR/HOMA‐IR) [26], DIo (IGI/fasting insulin) [27], DI30 (INSR30 × ISIc) and DI120 (INSR120 × ISIc), were used to assess β‐cell function corrected for insulin sensitivity [25].

### 2.3 GWAS Genotype Data

The genotype data utilized in our GWAS were derived from the Infinium Asian Screening Array‐24‐v1.0 as previously described [19]. In the data cleaning process, samples were excluded based on several criteria: absence of phenotype data, relationship status of second‐degree relatives or closer with an identity by descent (IBD) exceeding 0.2, DNA of suboptimal quality (manifested by a call rate below 90%), and inconsistencies in sex data. Furthermore, SNPs were excluded if they satisfied one or more of the ensuing stipulations: (1) location on a sex chromosome, (2) minor allele frequency (MAF) under 0.01, (3) call rate below 95%, and (4) Hardy–Weinberg equilibrium (HWE) test *p‐*value < 1.0 × 10^−6^. Genotype imputation was performed via the TOPMed online server, using Eagle v2.4 [28] for haplotype phasing and the TOPMed reference panel (Version r2) [29], which comprises 97 256 samples and 308 107 085 variants. The imputation processes were performed via Minimac software (v4), with all genetic variants being aligned to GRCh38 coordinates. Poorly imputed SNPs with imputation quality score *r* ^2^ < 0.3 and SNPs located on sex chromosomes were excluded from the subsequent analyses.

### 2.4 Statistical Analysis

In the analysis examining the associations between smoking status and indicators of insulin sensitivity and islet β‐cell function, a multiple linear regression model was utilized, adjusting for potential confounders, including gender, age, BMI, systolic blood pressure (SBP), diastolic blood pressure (DBP), Cr, HDL‐C, LDL‐C, TC, TG, history of hypertension, and CVD.

Considering the established association between insulin sensitivity and β‐cell function [30], we further performed a mediation analysis with smoking as the independent variable, insulin sensitivity index (ISIc) as the mediator, and beta cell function as the dependent variable, adjusting for the covariates mentioned above. Subsequent stratified analyses were conducted across the three genotype categories and the two categories of smoking status. In the association analysis between genotype and phenotype, the model was adjusted for potential confounders, and the same adjustments were applied in the stratified analysis of the association between smoking status and phenotypes.

### 2.5 Single Variant Association and Gene‐Smoking Interaction Analysis

Single‐variant association analyses were conducted utilizing REGENIE v3.2.6, a machine learning‐based approach designed to fit whole‐genome regression models for both quantitative and binary phenotypes [31]. The gene–environment interaction analysis was conducted employing the interaction function in REGENIE. Quantitative traits were rank‐based inverse normal transformed (INT). Association studies were performed in the discovery set (NJ cohort) and validation set (JR cohort), separately. In the association analyses, we adjusted for covariates, including age, gender, BMI, and the top ten principal components (PCs). A meta‐analysis was then performed to summarize the results between the discovery and replication sets for single variants using the METAL software [32]. The associations were considered significant if the variants met the following three criteria simultaneously: (1) *p* ≤ 1 × 10^−6^ in the discovery set, (2) *p* ≤ 0.05 in the validation set, and (3) *p* ≤ 5 × 10^−8^ in the meta‐analysis. Manhattan plots were visualized using the CMplot package [33].

### 2.6 Expression Quantitative Trait Locus Colocalization and Chromatin Interaction Analysis

We employed the 3DSNP tool (https://omic.tech/3dsnpv2), a database that offers the three‐dimensional interactions between SNPs and other genes to obtain functional annotations and expression quantitative trait locus (eQTL) of significant variants [34]. To perform colocalization analysis, we used a novel implementation of coloc called xQTLbiolinks across multiple tissues [35]. We investigated the locus by considering 1 Mb upstream and downstream of the sentinel SNP, which represents the most prominent signal in a specific genome region. PP.H4 > 0.7 was set as the threshold for shared genetic effects between the two traits, optimizing sensitivity and specificity to ensure robust signal detection and consistency in complex datasets. Additionally, we conducted chromatin interaction analysis on the variants using data from the 3DIV database (https://kobic.kr/3div/) [36]. This analysis specifically targeted a 2 Mb region around the variants within human pancreatic tissue, focusing on potential chromatin interactions within this area.

## 3 Results

### 3.1 General Characteristics of Nonsmokers and Smokers

We recruited 4808 and 508 participants with NGT in the NJ cohort (3817 nonsmokers and 991 smokers) and the JR cohort (364 nonsmokers and 144 smokers), respectively. Smokers were more likely to be male and to have higher BMI, SBP, DBP, Cr, and TG (beta > 0, *P*_NJ_ or *P*_JR_ < 0.05), as well as lower HDL‐C and TC (beta < 0, *P*_NJ_ or *P*_JR_ < 0.05) (Table 1). Further excluding those with missing DNA samples, those failing the DNA quality check, and genetic data quality control, 1377 individuals in the NJ cohort were utilized for the discovery phase of genetic and environmental interaction association analysis, while 485 individuals in the JR cohort were employed for the validation phase (Figure S1).

**TABLE 1:** Baseline characteristics of nonsmokers and smokers.

	Nonsmoker	Smoker	*p*
Age (years)			
NJ cohort	54.83 (9.05)	55.22 (9.20)	0.229
JR cohort	37.24 (10.89)	42.67 (11.87)	< 0.001
Male, *n* (%)			
NJ cohort	600 (15.7)	895 (90.3)	< 0.001
JR cohort	110 (30.2)	141 (97.9)	< 0.001
BMI (kg/m^2^)			
NJ cohort	23.56 (3.07)	24.20 (2.96)	< 0.001
JR cohort	23.23 (3.21)	25.15 (3.33)	< 0.001
SBP (mmHg)			
NJ cohort	123.83 (16.38)	126.47 (16.16)	< 0.001
JR cohort	117.65 (13.94)	128.09 (15.09)	< 0.001
DBP (mmHg)			
NJ cohort	75.53 (10.16)	79.04 (10.61)	< 0.001
JR cohort	79.12 (9.07)	85.25 (10.06)	< 0.001
Serum Creatinine (μmol/L)			
NJ cohort	64.74 (20.56)	76.55 (17.12)	< 0.001
JR cohort	59.41 (12.02)	73.17 (9.66)	< 0.001
HDL‐C (mmol/L)			
NJ cohort	1.39 (0.35)	1.22 (0.31)	< 0.001
JR cohort	1.58 (0.31)	1.37 (0.35)	< 0.001
LDL‐C (mmol/L)			
NJ cohort	2.78 (0.75)	2.74 (0.74)	0.138
JR cohort	2.89 (0.75)	3.09 (0.85)	0.014
Total cholesterol (mmol/L)			
NJ cohort	4.84 (1.00)	4.66 (0.95)	< 0.001
JR cohort	4.69 (0.90)	4.73 (1.00)	0.679
Triglycerides (mmol/L)			
NJ cohort	1.32 (0.86)	1.55 (1.00)	< 0.001
JR cohort	1.13 (0.86)	1.52 (1.33)	0.001
Fasting plasma glucose (mmol/L)			
NJ cohort	5.32 (0.35)	5.40 (0.35)	< 0.001
JR cohort	5.46 (0.34)	5.56 (0.34)	0.003
Plasma glucose of 30 min (mmol/L)			
NJ cohort	8.50 (1.49)	8.91 (1.46)	< 0.001
JR cohort	8.38 (1.36)	9.05 (1.55)	< 0.001
Plasma glucose of 120 min (mmol/L)			
NJ cohort	6.20 (0.97)	6.01 (1.05)	< 0.001
JR cohort	5.84 (1.02)	5.81 (1.10)	0.787
Fasting serum insulin (μU/mL)			
NJ cohort	9.96 (7.40, 13.50)	10.32 (7.64, 14.19)	0.065
JR cohort	11.6 (8.84, 14.95)	10.80 (7.88, 14.03)	0.143
Serum insulin of 30 min (μU/mL)			
NJ cohort	58.34 (38.96, 90.45)	56.75 (35.3, 91.73)	0.176
JR cohort	74.80 (49.40, 107.50)	67.15 (44.98, 102.75)	0.275
Serum insulin of 120 min (μU/mL)			
NJ cohort	42.85 (27.61, 66.48)	36.89 (22.57, 61.31)	< 0.001
JR cohort	45.60 (31.55, 66.90)	38.25 (22.70, 60.83)	< 0.001
HbA1c (%)			
NJ cohort	5.64 (0.38)	5.64 (0.54)	0.878
JR cohort	5.29 (0.33)	5.44 (0.31)	< 0.001
History of CVD, *n* (%)			
NJ cohort	180 (4.7)	35 (3.5)	0.128
JR cohort	5 (1.4)	8 (5.6)	0.017
History of Hypertension, *n* (%)			
NJ cohort	670 (17.6)	208 (21.0)	0.014
JR cohort	19 (5.2)	18 (12.5)	0.008
Disorder of lipid metabolism, *n* (%)			
NJ cohort	274 (7.2)	72 (7.3)	0.980
JR cohort	21 (5.8)	11 (7.6)	0.563
Family history of diabetes, *n* (%)			
NJ cohort	509 (13.3)	137 (13.8)	0.726
JR cohort	44 (12.1)	17 (11.8)	1.000

*Note:* Data are expressed as mean (SD), median (interquartile range) or *n* (%). Two‐sample *t*‐test, wilcoxon test or *χ* ^2^ test were used to assess differences between smokers and nonsmokers. NJ cohort included 3817 nonsmokers and 991 smokers; JR cohort included 364 nonsmokers and 144 smokers.

### 3.2 Associations of Smoking With Insulin Sensitivity and β‐Cell Function

**FIGURE 1:**
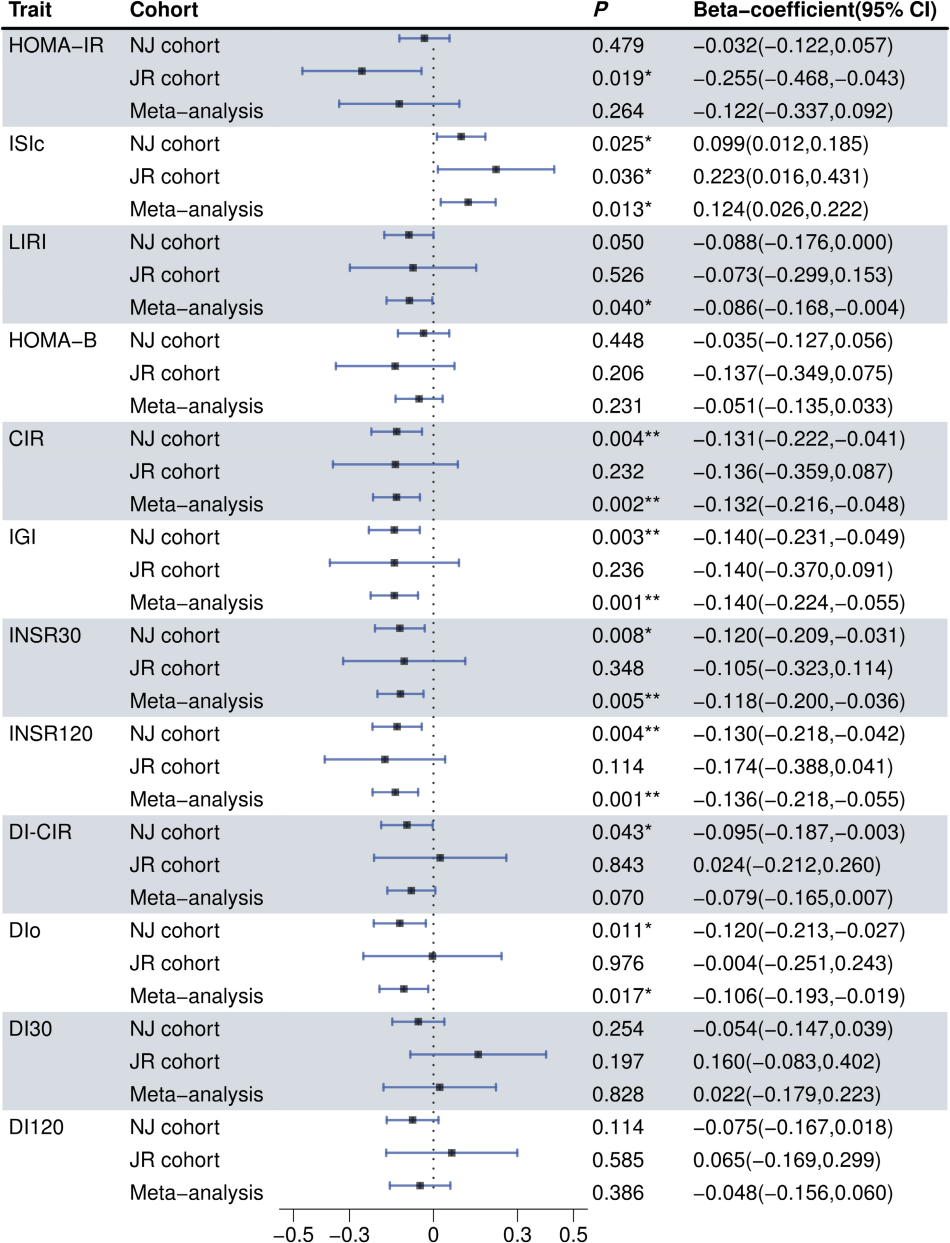
Association of smoking status with insulin sensitivity and islet β‐cell function. All dependent variables were rank‐based inverse normal transformed, and multiple linear regression analysis was conducted to adjust for gender, age, BMI, SBP, DBP, Cr, HDL‐c, LDL‐c, TC, TG, history of hypertension and history of CVD. The horizontal lines denote the 95% confidence intervals of the regression coefficients.

We explored the relationship between smoking status and both insulin sensitivity and islet β‐cell function within NGT participants. After performing multivariable linear regression and adjusting for previously reported potential confounders related to insulin sensitivity and β‐cell function, we found a positive correlation between the insulin sensitivity index ISIc and smoking status in NJ (beta = 0.099, *P*_NJ_ = 0.025), JR cohort (beta = 0.223, *P*_JR_ = 0.036) and meta analysis (beta = 0.124, *P*_meta_ = 0.013) (Figure 1).

In contrast, regarding the relationship between smoking and islet β‐cell function, smoking status was negatively correlated with the insulinogenic index (IGI) (beta = −0.140, *P*_meta_ = 0.001). Early phase insulin secretion INSR30 (beta = −0.118, *P*_meta_ = 0.005) and total insulin secretion INSR120 (beta = −0.136, *P*_meta_ = 0.001) also presented a similar correlation with smoking status. However, no significant correlation was observed between smoking status and either early‐phase (DI30) or total disposition indices (DI120). Given that DI accounts for insulin sensitivity, this implies that the effect of smoking on the secretory function of islet β‐cells could be mediated through alterations in insulin sensitivity (Figure 1). Mediation analysis further supported this observation. The insulin sensitivity index (ISIc) accounted for 34.02%, 28.96%, 61.96%, and 59.50% of the effect of smoking on four β‐cell function indicators: CIR, IGI, INSR30, and INSR120, respectively in the NJ cohort (Table S1).

### 3.3 Gene‐Smoking Interaction With Insulin Sensitivity and β‐Cell Function

**FIGURE 2:**
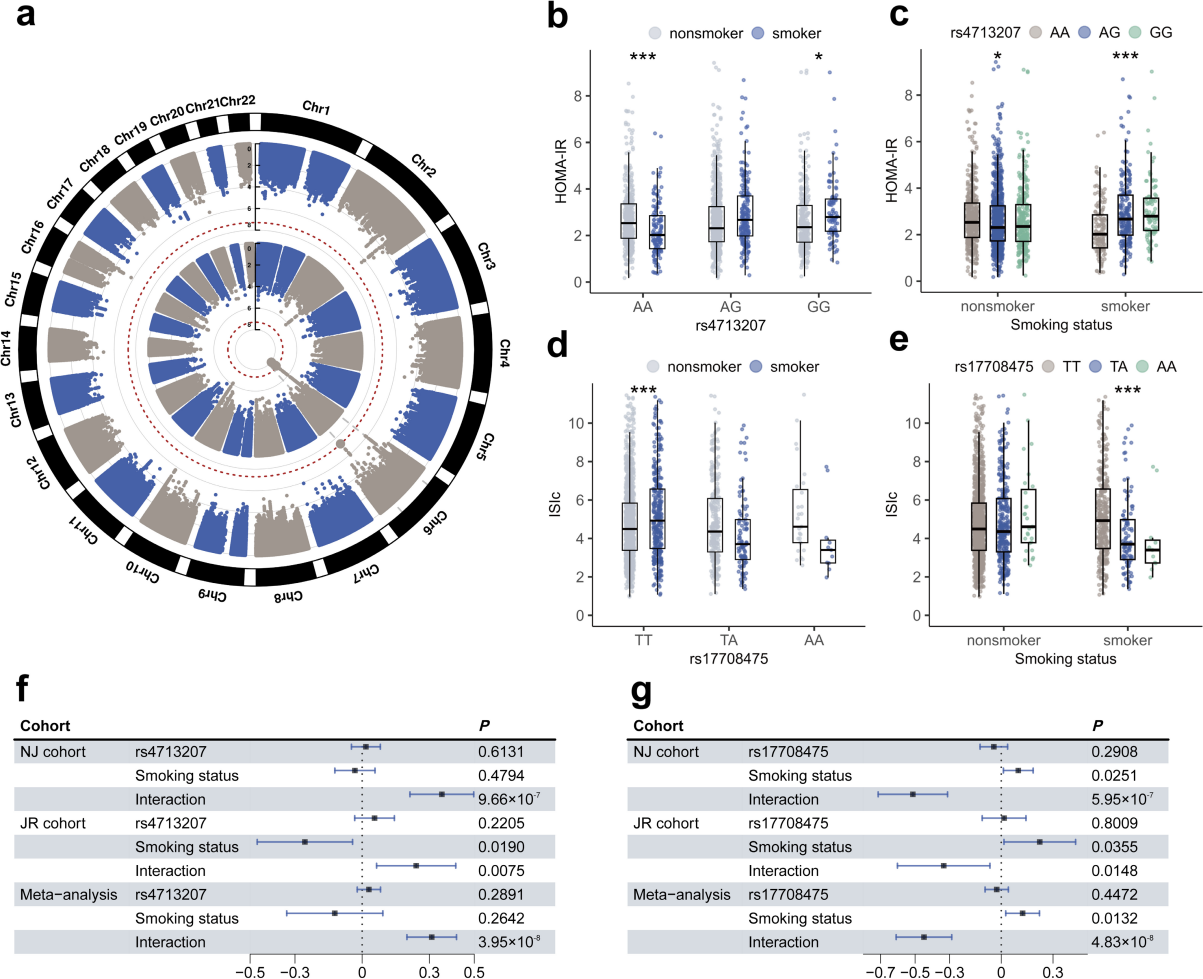
Gene‐smoking interaction with insulin sensitivity. (a) Circular‐manhaton plot of HOMA‐IR (inner layer) and ISIc (outer layer) in meta‐analysis. (b) The effect of smoking on HOMA‐IR under different genotypes of rs4713207. Gender, age and BMI were adjusted in the multivariate regression. * *P* < 0.05, ** *P* < 0.005, *** *P* < 0.0005. (c) The effect of rs4713207 on HOMA‐IR under different smoking status. Gender, age and BMI were adjusted in the multivariate regression. (d) The effect of smoking on ISIc under different genotypes of rs17708475. (e) The effect of rs17708475 on ISIc under different smoking status. (f) The main and interaction effects of rs4713207 and smoking on HOMA‐IR. (g) The main and interaction effects of rs17708475 and smoking on ISIc.

We subsequently performed interaction analyses to investigate the effect of gene‐smoking interactions on insulin sensitivity and β‐cell function on a genome‐wide scale, utilizing both the discovery (NJ cohort) and validation (JR cohort) sets. A total of ten SNPs in three regions were identified as significant for potential interaction (*p* ≤ 1 × 10^−6^ in the discovery set, *p* ≤ 0.05 in the validation set, with consistent effect directions and *P*_meta_ < 5 × 10^−8^) (Tables S2 and S3). One of these regions, located on chromosome 6p22.1 (closest annotated gene *OR14J1*), exhibited interaction effects with smoking on insulin resistance (HOMA‐IR) (Table 2, Figure 2a,f). We then conducted stratified analyses on the variant rs4713207, which exhibited the highest functional score in the 6p22.1 region according to 3DSNP (Table 2, see footnote “^e^”) to clarify the differential effects of various genotypes and smoking status on HOMA‐IR. To evaluate the clinical significance and ensure statistical robustness, we report the coefficients, confidence intervals, and *p*‐values before the inverse normal transformation of the phenotype, as well as the *P*_INT_ after the transformation. Among individuals with the wild‐type (AA genotype) of rs4713207, smokers have a lower HOMA‐IR compared to nonsmokers (beta = −0.80, 95% CI = [−1.26, −0.35], *p* = 6.17 × 10^−4^, *P*_INT_ = 2.15 × 10^−5^). In contrast, smokers showed higher HOMA‐IR, with the effect being statistically significant in the homozygous (GG genotype) group (beta = 0.36, 95% CI = [−0.06, 0.78], *p* = 0.094, *P*_INT_ = 0.022). No statistically significant effect was observed in the heterozygous (AG genotype) group (Figure 2b). Additionally, after stratifying by smoking status, we found that the effect of genotype on HOMA‐IR was more pronounced in smokers (beta = 0.39, 95% CI = [0.20, 0.58], *p* = 4.90 × 10^−5^, *P*_INT_ = 1.95 × 10^−7^), while the HOMA‐IR across different genotypes is nearly equal in nonsmokers (Figure 2c).

**TABLE 2:** Top scoring variants affecting insulin sensitivity and islet function in gene‐smoking interactions.

Trait	SNP	Position^a^	Cytoband	Gene^b^	Allele^c^	EAF (NJ; JR)^d^	Score^e^	*P*_NJ_	*P*_JR_	*P*_meta_
Insulin sensitivity										
HOMA‐IR	rs4713207	29 296 878	6p22.1	*OR14J1*	A_G	0.47; 0.44	4.46	9.66 × 10^−7^	7.46 × 10^−3^	3.95 × 10^−8^
ISIc	rs17708475	124 617 296	6q22.31	*NKAIN2*	T_A	0.13; 0.13	1.02	5.95 × 10^−7^	1.48 × 10^−2^	4.83 × 10^−8^
β‐cell function										
DI‐CIR	rs201613	10 658 553	17p13.1	*MYH3*	A_T	0.66; 0.68	1.40	7.54 × 10^−8^	1.20 × 10^−2^	1.05 × 10^−8^

^a^ Genome position was based on GRCh38 coordinate.

^b^ Nearest coding gene.

^c^ Reference allele/effect allele.

^d^ Effect allele frequency.

^e^ Score of functionality from 3DSNP.

**FIGURE 3:**
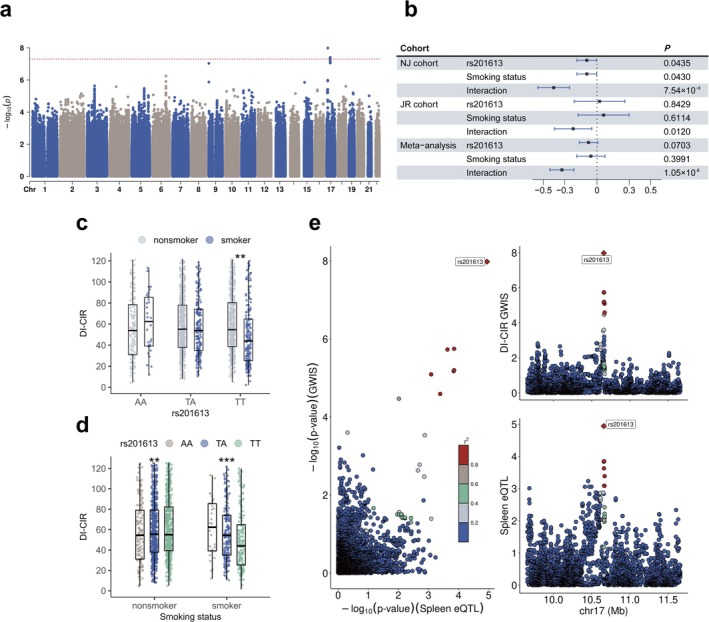
Gene‐smoking interaction with islet β‐cell function. (a) Manhaton plot of DI‐CIR in meta‐analysis. (b) The main and interaction effects of rs201613 and smoking on DI‐CIR. (c) The effect of smoking on DI‐CIR under different genotypes of rs201613. Gender, age and BMI were adjusted in the multivariate regression. * *P* < 0.05, ** *P* < 0.005, *** *P* < 0.0005. (d) The effect of rs201613 on DI‐CIR under different smoking status. Gender, age and BMI were adjusted in the multivariate regression. (e) Locus Zoom plots for rs201613 in the DI‐CIR GWIS meta‐analysis and spleen eQTL.

The additional two top scoring variants, rs17708475 (*P*_meta_ = 4.83 × 10^−8^, an intronic variant in *NKAIN2* on 6q22.31) and rs201613 (*P*_meta_ = 4.83 × 10^−8^, an intronic variant in *MYH3* on 17p13.1), were found to be associated with insulin sensitivity (ISIc) and β‐cell function indicator (DI‐CIR) (Table 2, Figure 2a,g, Figure 3a), respectively. In smokers, the association between rs17708475 and ISIc is significant (beta = −1.12, 95% CI = [−1.57, −0.67], *p* = 1.69 × 10^−6^, *P*_INT_ = 1.82 × 10^−7^), demonstrating a decrease in insulin sensitivity as the number of risk alleles increased, while this relationship was not observed in nonsmokers (Figure 2d, Figure 2e). For rs201613, among participants with homozygotes (TT genotype), smoking was associated with lower DI‐CIR (beta = −21.84, 95% CI = [−91.48, 47.81], *p* = 0.54, *P*_INT_ = 0.0027). However, in both the heterozygous (AT genotype) and wild‐type (AA genotype) groups, the effect of smoking did not reach statistical significance (Figure 3b–d).

### 3.4 Expression Quantitative Trait Locus and Colocalization Analysis

According to the 3DSNP database, we detected 25 eQTL‐significant genes (false discovery rate FDR ≤ 0.05) for rs4713207. For example, under the additive model, each copy of the G allele compared with the A allele was associated with decreased expression of *ZNF311* in thyroid (beta = −0.18, *p* = 6.91 × 10^−8^), adipose visceral omentum (beta = −0.14, *p* = 1.96 × 10^−5^), breast mammary tissue (beta = −0.14, *p* = 2.86 × 10^−5^), and spleen (beta = −0.24, *p* = 8.22 × 10^−5^). In addition, there was higher *HCG4* expression in esophagus mucosa (beta = 0.28, *p* = 7.15 × 10^−7^), thyroid (beta = 0.24, *p* = 1.18 × 10^−5^), coronary artery (beta = 0.32, *p* = 6.11 × 10^−5^) and pancreas for the G allele compared with the A allele (Table S4). Moreover, chromatin interaction analysis from 3DIV showed that rs4713207 potentially interacts with *ZSCAN26* in pancreas tissue (Table S5). However, no eQTL signals were detected for rs17708475. For rs201613, each copy of the T allele, compared with the A allele, was associated with increased expression of *CTC‐297 N7.1* in spleen tissue (beta = 0.14, *p* = 1.14 × 10^−5^), a tissue typically related to metabolic diseases. Chromatin interaction analysis indicated a potential interaction between rs201613 and the myosin gene *MYH13* (Table S6).

We then performed colocalization analyses for rs4713207 and rs201613, respectively, using xQTLbiolinks based on the significant eQTLs detected in 3DSNP. The analysis identified two distinct colocalization signals within the region 6p22.1. Notably, the posterior probability of a shared causal variant for *ZNF311* expression in spleen and insulin resistance reached 74%; for *HCG4* expression in pancreas and insulin resistance, it was 70%, indicating that the interaction effect of this region with smoking on insulin resistance may be mechanistically mediated by these two genes. The expression of *CTC‐297 N7.1* in spleen exhibited a high posterior probability of colocalization with disposition index (PP.H4.abf = 0.97), and rs201613 was likely to serve as a shared causal variant for both traits (SNP.PP.H4 = 1.00) (Table S7, Figure 3e).

## 4 Discussion

In the present study, we performed a two‐stage GWIS to identify potential interactions between SNPs and smoking that may impact insulin sensitivity and islet β‐cell function, using data from NGT participants from two cohort studies. We identified three loci exhibiting significant interactions with smoking, including two associated with insulin sensitivity and one with islet β‐cell function.

The relationship between smoking and the underlying mechanisms of T2D remains controversial. A study among Chinese adult males found no association between active smoking and insulin sensitivity among individuals with NGT [37]. Another study conducted in a non‐diabetic Chinese cohort reported that current smokers have slightly higher insulin sensitivity than nonsmokers [38]. Conversely, some epidemiological studies suggest that smoking contributes to impaired glucose tolerance and may induce insulin resistance [39, 40]. Additionally, variable effects of nicotine on insulin sensitivity have been demonstrated in experimental research. Long‐term low‐dose nicotine treatment has been reported to impair insulin sensitivity, while high‐dose nicotine treatment may enhance it [41, 42]. These inconsistencies may result from variations in genetic susceptibility, smoking patterns, ethnicity, and baseline glucose metabolic status [43, 44, 45]. Given that hyperglycemia‐induced glucotoxicity can impair islet β‐cell function [46], we specifically focused on participants with NGT to minimize the potential confounding effects of glucose metabolism factors.

Our findings indicated that smokers displayed slightly higher insulin sensitivity, as measured by ISIc, compared to nonsmokers. Furthermore, smokers showed diminished islet β‐cell insulin secretion capacity, consistent with previous research. Notably, this difference in β‐cell function disappeared after adjusting for insulin sensitivity, suggesting that smoking may primarily affect insulin secretion through its impact on insulin sensitivity. Mediation analysis further confirmed this indirect effect. Genetic factors and gene–environment interaction are known to play a significant role in the development of metabolic diseases [47]. Prior research has indicated that smoking status accounted for 22% of the gene–environment variance in HOMA‐B [48], suggesting that gene–smoking interactions can provide additional biological pathways contributing to the heterogeneity observed in previous research.

We observed an interaction between smoking status and insulin resistance in a large LD block on 6p22.1, near the *OR14J1* gene, where the effects of alleles on insulin resistance varied depending on smoking status. For rs4713207, smokers exhibited a lower insulin resistance index compared to nonsmokers in the wild‐type group, whereas in the homozygous group, smokers showed a higher level of insulin resistance. In the heterozygous group, the opposing effects of the two alleles on smoking appeared to counterbalance each other, leading to no observed differences between smokers and nonsmokers. Since the allele frequencies are relatively similar and the heterozygous genotype is most prevalent in the population, it is not surprising that the overall impact of smoking on insulin resistance was not detectable. Rs4713207 is implicated in the expression of several genes in metabolically relevant tissues, including pancreas, muscle, and spleen. The eQTL colocalization analysis indicated that this region may influence insulin sensitivity in specific smoking status by affecting the expression of *HCG4* in the pancreas and *ZNF311* in the spleen. This could explain the difficulty in observing the main effects of these genes. *ZNF311* and *HCG4* have been previously associated with rheumatoid arthritis and preeclampsia, conditions that are known to be associated with a higher prevalence of insulin resistance compared to the general population [49, 50]. Additionally, *OR14J1* encodes an olfactory receptor that interacts with odorant molecules in the nose, triggering neuronal responses that contribute to olfactory perception. Similar to smoking, damage to OR genes can impair the olfactory system, and accumulating evidence has suggested that olfactory dysfunction is associated with insulin resistance and other metabolic disorders [51, 52, 53]. The results from the 3DIV revealed an interaction between *OR14J1* and *ZSCAN26* in pancreatic tissue. An integrative epigenome‐wide association study has shown that CpG sites within *ZSCAN26* are associated with lower insulin resistance [54].

Additionally, the variant rs17708475 within the *NKAIN2* gene was found to interact with smoking, influencing the index of insulin sensitivity (ISIc). *NKAIN2* is a protein‐coding gene that encodes a transmembrane protein, which interacts with Na+/K+ ATPase. Smoking induces oxidative stress by increasing the production of reactive oxygen species (ROS), which in turn activates the Na+/K+ ATPase signaling cascade within cells [55]. The impaired activity of Na+/K+ ATPase exacerbates oxidative stress through a feedback loop, thereby promoting insulin resistance [56]. Experimental studies have provided evidence that the Na+/K+ ATPase pathway is a therapeutic target for insulin resistance [57]. Consequently, the observed higher insulin sensitivity in smokers with the wild‐type of rs17708475 may be due to the regulatory role of *NKAIN2* on Na+/K+ ATPase.

In the context of β‐cell function, we identified an interaction between the intronic variant rs201613 in the gene *MYH3* on 17p13.1 and smoking, influencing the disposition index (DI‐CIR). Similar effects were observed for other disposition indices (DI30 and DI120), although these did not reach the significance threshold. The adverse effect of smoking on β‐cell insulin secretion was evident only in individuals carrying the wild‐type genotype. eQTL colocalization analysis suggested that this phenomenon may be associated with the regulatory activity of the long non‐coding RNA gene *CTC‐297N7.1*. *MYH3* is a core regulator of embryonic skeletal muscle development and mediates myofibril assembly. Experimental evidence has shown that cigarette smoke exposure can result in downregulation of *MYH3* transcription [58, 59]. Although *MYH3* has not been directly linked to metabolic diseases, numerous studies have demonstrated its role in modulating TGF‐β signaling and affecting ATPase activity, which may contribute to conditions such as joint contractures and skeletal muscle abnormalities [60, 61]. Given that skeletal muscle serves as a sort of buffer against hyperglycemia after a glucose load, maintaining muscle mass is crucial for preventing the onset and progression of prediabetes to T2D [62]. In addition, the heterogeneity across studies may be explained by the differing frequencies of pathogenic *MYH3* variants among populations [63].

The functional exploration of the aforementioned genes provides evidence supporting the existence of gene‐smoking interactions. Our study has several limitations. First, although this study included a population of Han ethnicity individuals from Jiangsu Province, China, with good genetic homogeneity, further research is needed to determine whether these associations are applicable to other ethnic groups. Second, to exclude the effects of glucotoxicity on insulin sensitivity and β‐cell function, this study focused on the NGT population. However, diabetic individuals may show higher genetic susceptibility and variance in glucose metabolism disorders, potentially revealing additional significant SNPs. Therefore, future research in diabetic populations could further validate and refine these findings. Third, the limited sample size, along with the absence of data such as pack‐years and cotinine levels, constrained the analysis in this study, thereby hindering the additional detection of biologically relevant associations.

In conclusion, our study highlights potential interactions between smoking and genetics. The impact of smoking on insulin sensitivity and islet β‐cell function may vary considerably across different genotypes. Our findings provide novel insights into the heterogeneity observed in the relationship between smoking and metabolic phenotypes, contributing to personalized risk assessment and supporting strengthened interventions for high‐risk genetic groups.

## Disclosure

The authors have nothing to report.

## Conflicts of Interest

The authors declare no conflicts of interest.

## Supporting information


Figure S1.

Table S1.
