# Supplementary figures and images for: Gene‐Smoking Interaction in Insulin Sensitivity and β‐Cell Function Among Normal Glucose Tolerance Individuals
Source: J Diabetes. 2025 Jul 28;17(7):e70131. doi: 10.1111/1753-0407.70131 (PMC12301937; doi:10.1111/1753-0407.70131)

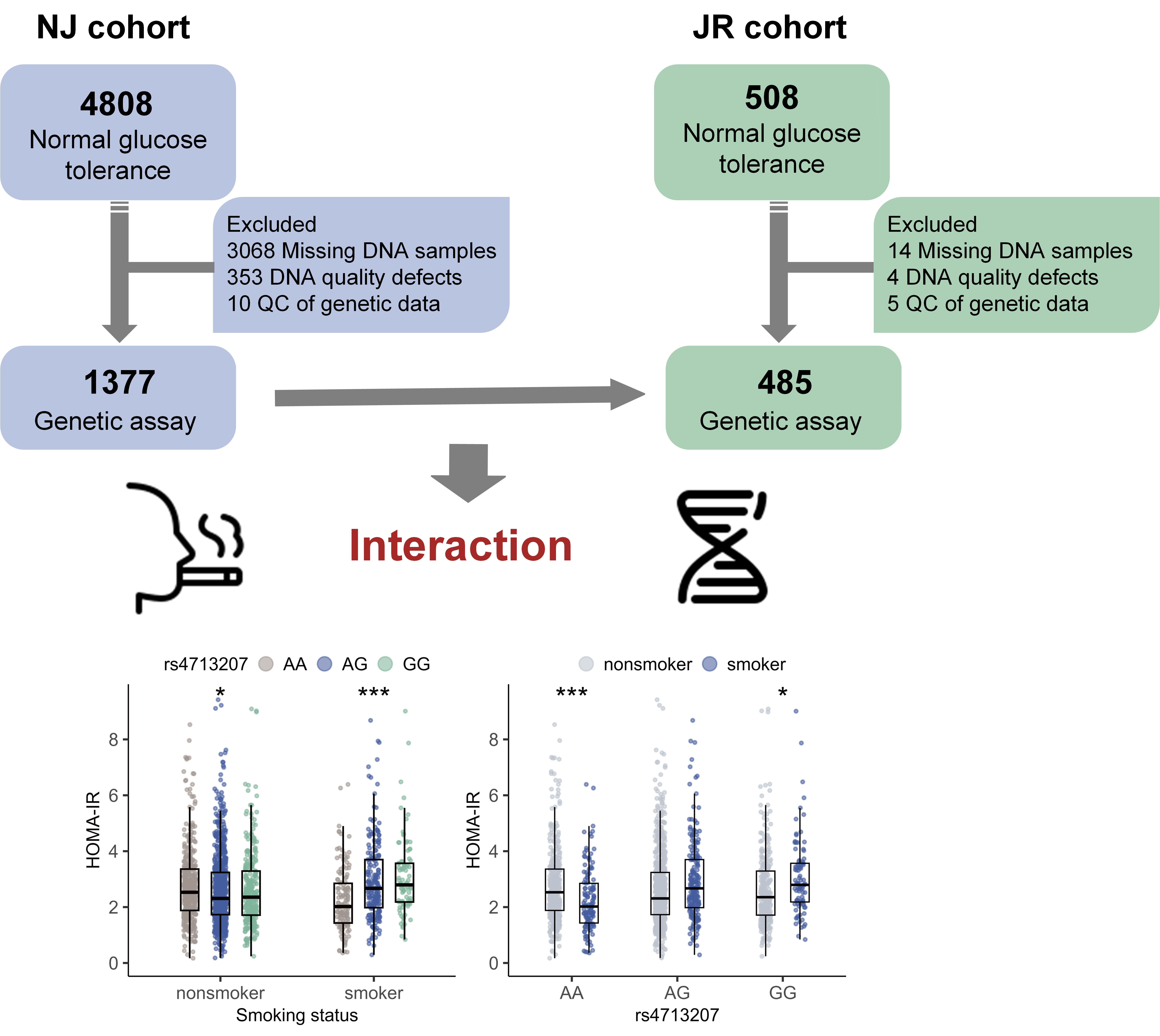

Supplement: Supplementary file 1 — Figure S1. [file JDB-17-e70131-s002.jpg]
